# The effectiveness of problem-based learning in gynecology and obstetrics education in China: A meta-analysis of randomized controlled trials

**DOI:** 10.1097/MD.0000000000024660

**Published:** 2021-03-05

**Authors:** Siwei Bi, Ruiqi Liu, Jingyi Li, Jun Gu

**Affiliations:** a Department of Burn and Plastic Surgery, West China Hospital; b West China School of Medicine; c Department of Cardiovascular Surgery, West China Hospital, Sichuan University, Chengdu, Sichuan, People's Republic of China.

**Keywords:** gynecology and obstetrics, meta-analysis, randomized controlled trials

## Abstract

**Background:**

A meta-analysis was conducted to assess the effectiveness of problem-based learning (PBL) in gynecology and obstetrics education in China.

**Methods:**

English and Chinese databases were systematically searched for eligible studies that compared the effects of PBL and traditional teaching methods measuring theoretical knowledge, student satisfaction, clinical operations, and clinical practice scores in gynecology and obstetrics education in China. The authors restricted included studies to randomized controlled trials and performed a meta-analysis. Standardized mean difference (SMD) and risk ratio with 95% confidence interval (CI) were estimated

**Results:**

A total of 38 randomized controlled trials with 3005 participants were included. Compared with traditional teaching group, the PBL group significantly increased theoretical knowledge scores (SMD: 3.17, 95% CI: 2.28, 4.07), student satisfaction (risk ratio: 1.29, 95% CI: 1.16, 1.43), clinical operations (SMD: 1.15, 95% CI: 0.93, 1.37) and clinical practice (SMD: 2.17, 95% CI: 3.63, 2.71).

**Conclusion:**

The current research shows that PBL in gynecology and obstetrics education in China is more effective than the traditional teaching in enhancing theoretical knowledge, student satisfaction, clinical operations, and clinical practice scores. However, more delicate-designed studies on this topic are needed in the future to validate these results.

## 1 Introduction

Problem-based learning (PBL), an innovative approach in medical education, was originally introduced at McMaster University in the late^[1]^ 1960s. It is a student-centered, teacher-directed education method that uses real problems as the context of learning and acquires knowledge actively^[2]^ and innovatively. Nowadays, it has been widely used in many training programs under various circumstances.^[3]^ As shown in the previous literature,^[4–6]^ PBL students would sometimes outperform the students with traditional teaching methods, but sometimes did not. Besides, the results would also be different when considering the different outcomes such as knowledge- and skills-related outcomes.

As 1 expert review^[7]^ pointed out, the changing of generations means the corresponding modification of the teaching method in education. The online PBL has already been an example of e-learning.^[8]^ Moreover, due to the recent improvements in the education approach in China, an increasing number of training programs chose PBL as one of the experimental educational methods^[3,9]^ in multiple majors. However, the implementation of PBL in medical education is still a novel teaching method in China since the different educational system and cultural background. Most Chinese students have not received this kind of education since the beginning of primary school their primary school.^[10]^ There is no published study indicating whether PBL is superior to traditional teaching methods in obstetrics and gynecology education or not. The aim of the current meta-analysis was to investigate the effectiveness of PBL compared with the traditional teaching methods in Chinese obstetrics and gynecology education focusing on theoretical knowledge, student satisfaction, clinical operation, and clinical practice.

## 2 Methods

### 2.1 Data sources

The following English and Chinese databases were searched systematically: China National Knowledge Infrastructure, Wanfang Data (WAN-FANGDATA), CQVID, PubMed, EMBASE, and Cochrane Database using the following terms: (PBL OR [problem-based learning]) AND (obstetrics and gynecology). The publishing dates of including articles were from January 1, 2015 to the searching date: February 22, 2020 without any language restriction. Reference lists of primary articles were reviewed for more extra literature. The present study does not need ethical approval since all analyses were based on previously published studies,

### 2.2 Inclusion criteria and study selection

Inclusion criteria are as follows:

(1) target population: obstetrics and gynecology medical students, interns or resident doctors in China;
(2) study design: controlled trials in obstetrics and gynecology education;
(3) interventions: PBL teaching in the experimental group and traditional teaching in the control group;
(4) outcome measurements: student satisfaction, clinical operation score, theoretical knowledge score, and clinical practice scores.

Meanwhile, we excluded studies with insufficient data for calculating effect sizes. All of the titles and abstracts were reviewed independently by the 2 reviewers (SWB, JYL). Any differences were resolved through consensus, and if necessary, a senior reviewer (RQL) was consulted.

### 2.3 Data extraction and quality assessment.

This process was conducted independently by the 2 reviewers (SWB, JYL) by the Cochrane Collaboration for Systematic Reviews guidelines.^[11]^ Relevant data from the eligible studies were extracted including the 1st author's name, the published date, the study type, the number of participants, median age, duration of study, population, intervention, and outcome measurements. The methodologic quality of each study was evaluated based on the assessment of the following items: random sequence generation, allocation sequence concealment, blinding of participants and personnel, blinding of the outcome assessment, incomplete outcome data, selective reporting, and other biases. For each study, every item was rated as “low risk of bias,” “high risk of bias,” or “unclear risk of bias.”^[12]^

### 2.4 Subgroup analysis and statistical analysis

Standardized mean difference (SMD) for continuous outcomes, risk ratio for dichotomous outcomes with 95% confidence interval (CI), was calculated for each study. Studies were then pooled together using SMD as appropriate with 2-sided *P* < .05 considered as statistically significant. The *Q*-statistic was calculated to examine result heterogeneity among studies, and *P* < .10 was considered significant. The authors first used the fixed-effects model with the assumption that the included studies were homogenous with *P* > .10; otherwise, the random-effects model was applied. The *I*^2^ statistic was also calculated to efficiently test for the heterogeneity, with *I*^2^ < 25%, 25% to 75%, and >75% to represent a low, moderate, and high degree of inconsistency, respectively.^[13]^ Moreover, we ran influence^[14,15]^ analysis for each outcome in the random model to find out the contribution of each study to the pooled effect and overall heterogeneity. Influence analysis is based on the Leave-One-Out-method, in which we recalculate the results of our meta-analysis K−1 times, each time leaving out 1 study. (K equals to the number of included studies) This way, studies that influence the overall estimate of meta-analysis the most would be detected. Publication bias was examined in contour-enhanced^[16]^ funnel plots where 3 studies with most heterogeneity contributions were highlighted. After excluding these 3 most heterogenous studies, we conducted a subgroup analysis to detect the source of heterogeneity further based on the populations. The meta-analysis and illustrations were performed using R 3.6.2 with packages^[17–20]^: “gemtc,” “rjags,” and “dmetar,” and “ggplot2.”

## 3 Results

### 3.1 Search results

**Figure 1:**
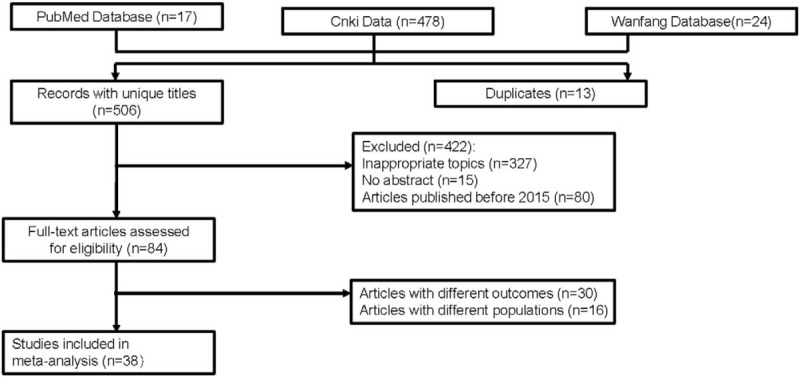
Flowchart for searching and identifying eligible studies.

The flowchart for the study selection process is shown in Figure 1. A total of 519 studies was selected from databases for further screening. We excluded 13 duplicated articles and 422 other articles because of inappropriate topics (n = 327), lack of abstract (n = 15), unanticipated target population (n = 16) and publishing restriction (n = 80). After assessing articles with full text, 30 studies were excluded since the different outcomes from our studies. In the end, a total of 38 controlled studies with 1494 participants in the PBL group and 1511 participants in traditional teaching groups were included for this meta-analysis.^[21–58]^

### 3.2 Study characteristics

The characteristics of included 38 studies are shown in Table 1. All of them were published in Chinese between 2015 and 2020 with an assessment of the effects of PBL compared with traditional teaching in obstetrics and gynecology courses. The sample sizes ranged from 31 to 218 with a median of 71. The majority of studies focused on the interns (n = 21) and 12 studies for medical students, 3 for resident doctors, 1 for foreign students, and 1 for master's degree students. We combined the foreign students with medical students for the following subgroup analysis. Twelve studies are missing duration data while 12 studies chose 12 months as the study duration. For bringing in as little bias as possible, we only included the studies with duration longer than 6 months in the meta-analysis (n = 21). The most frequent outcome is theoretical knowledge scores which was used to assess how well the students mastered the related theoretical knowledge. The scores in the clinical practical evaluate the students’ clinical practice including medical history collecting, physical examination, and case presentation. Measurements on how students perform during operation test and their satisfaction towards teaching are clinical operation scores and student satisfaction, respectively.

**Table 1:** The detailed baseline characteristics of all included studies.

Author	Publication time	Study type	Populations	M/F	No. of PBL group	Mean Age of PBL group	No. of traditional teaching group	Mean Age of traditional teaching group	Study Duration (mo)	Outcomes
HJ Chen^[21]^	2020	RCT	Resident Doctor	7/65	36	38.58	36	37.10	12	SS, CO, TK
WH Huang^[22]^	2019	RCT	Interns	15/55	35	23	35	21.9	13	CO, TK, CP
K Wang^[23]^	2019	RCT	Interns	8/92	50	20.4	50	21.5	26	TK, CP
HQ Wang^[24]^	2019	RCT	Interns	6/54	30	21.2	30	21.2	1	TK
YH Jiao^[25]^	2019	RCT	Interns	27/29	28	24.38	28	25.01	17	CO, TK
YD Yang^[26]^	2019	RCT	Medical students	38/42	40	20.12	40	20.52	12	TK, CP
L Li^[27]^	2019	RCT	Medical students	5/115	60	22.9	60	23.2	9	TK, CP
Y Li^[28]^	2019	RCT	Resident Doctor	34/66	50	22.47	50	21.06	24	SS, TK, CP
YJ Zhang^[29]^	2019	RCT	Interns	47/33	40	20.12	40	22.12	12	SS, CP
XP Shang^[30]^	2019	RCT	Medical students	44/47	29	20.17	62	20.34	NA	TK
Q Han^[31]^	2018	RCT	Interns	All female	22	22.5	22	22.5	12	SS, CO, TK
LS Guo^[32]^	2018	RCT	Medical students	NA	60	NA	60	NA	NA	SS, TK
HJ Zhen^[33]^	2018	RCT	Interns	All female	30	21.3	30	21.3	12	SS
LJ Zhen^[34]^	2018	RCT	Resident Doctor	17/17	18	25.26	16	25.05	24	SS, CO, TK
YY Wang^[35]^	2018	RCT	Interns	9/81	45	23.4	45	22.5	12	CO, TK
HX Wang^[36]^	2018	RCT	Interns	NA	30	21.6	30	21.1	3	CO, TK, CP
ZL Wang^[37]^	2018	RCT	Master	NA	19	NA	12	NA	NA	TK, CP
XM Shen^[38]^	2018	RCT	Medical students	40/44	44	20.6	42	20.8	NA	SS, CO, TK
X Li^[39]^	2018	RCT	Medical students	NA	50	NA	50	NA	NA	TK
FF Zhu^[40]^	2018	RCT	Interns	All female	40	NA	40	NA	NA	CO, TK
X Zhang^[41]^	2018	RCT	Medical students	NA	20	21.35	21	21.35	NA	SS, TK
JH Dang^[42]^	2018	RCT	Medical students	NA	28	NA	28	NA	NA	CO, TK
WZ Chen^[43]^	2017	RCT	Medical students	NA	30	NA	31	NA	NA	SS, TK
YY Wang^[44]^	2017	RCT	Interns	65/55	60	18	60	19	NA	SS, CO, TK
XF Tang^[45]^	2017	RCT	Interns	NA	30	NA	28	NA	9	CO, TK
Y Hua^[46]^	2017	RCT	Interns	54/78	66	23.3	66	23.5	12	TK
HY Liu^[47]^	2017	RCT	Medical students	35/25	30	20.31	30	20.2	4	SS, TK
WJ Hou^[48]^	2017	RCT	Interns	84/164	109	23.8	109	23.8	12	TK, CP
ZJ Gao^[49]^	2016	RCT	Interns	11/59	35	24.06	35	24.11	2	CO, TK
JH Han^[50]^	2016	RCT	Interns	All female	20	22.7	20	22.7	15	SS, CO
XL Wang^[51]^	2016	RCT	Medical students	26/54	40	24.3	40	24.3	12	SS, CO, TK
HX Wang^[52]^	2016	RCT	Medical students	NA	40	NA	39	NA	NA	TK
YH Li^[53]^	2016	RCT	Interns	12/86	49	22	49	22	39	CP
DY Zhu^[54]^	2016	RCT	Interns	28/32	30	NA	30	NA	NA	CO, TK
F He^[55]^	2016	RCT	Interns	2/58	30	NA	30	NA	12	SS, CO
MF Lin^[56]^	2015	RCT	Interns	NA	56	NA	52	NA	12	TK
CD Liu^[57]^	2015	RCT	Foreign students	21/39	30	25.31	30	25.41	6	TK
CY Mai^[58]^	2015	RCT	Interns	All female	35	22.5	35	21.8	12	CO, TK

### 3.3 Study quality assessment

**Figure 2:**
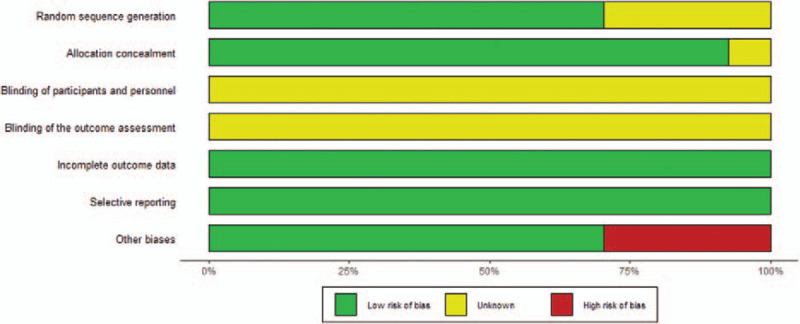
Summary of risk of bias in the included randomized controlled trials.

The summary risk of bias assessment of the 38 included studies was illustrated in Figure 2. The authors showed the results of each quality item as percentages across studies. Although all studies claimed randomized controlled trial (RCT)-designed, 16 studies are ambiguous about random sequence generation and all studies did not clarify the blinding of participants and personnel and blinding of the outcome assessment. We downgraded the 12 studies without clear definition of follow-up duration accordingly in the other bias section. All studies reported complete outcome data and were free of selective reporting.

### 3.4 Meta-analysis result for theoretical knowledge

**Figure 3:**
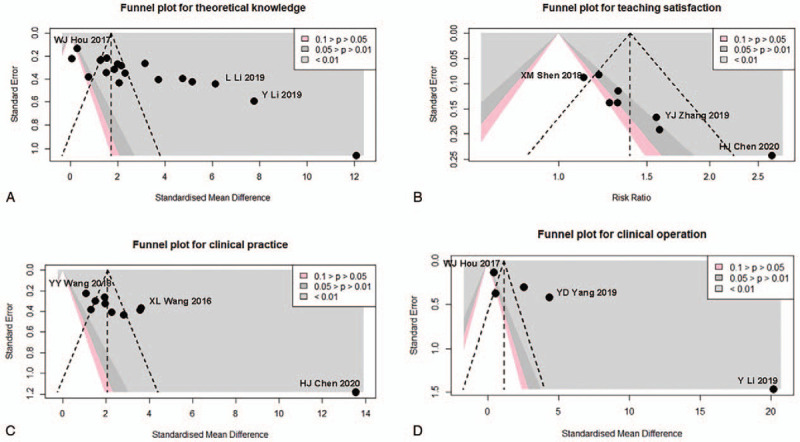
Contour-enhanced funnel plots. (A) Potential publication bias in the meta-analysis for theoretical knowledge scores. (B) Potential publication bias in the meta-analysis for student satisfaction. (C) Potential publication bias in the meta-analysis for clinical practice. (D) Potential publication bias in the meta-analysis for clinical operation. Each dot represents 1 study and the studies which were excluded from further meta-analysis are annotated with authors’ name.

**Figure 4:**
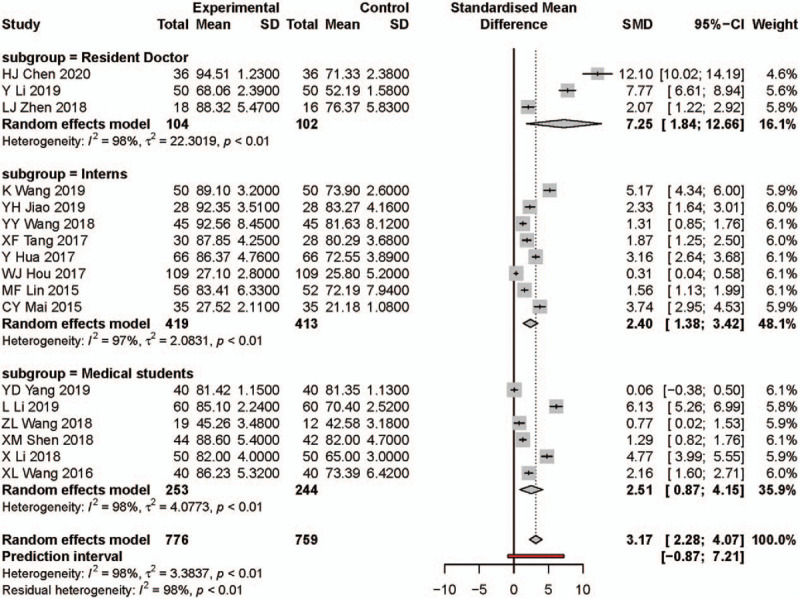
Forest plot and subgroups analysis results for theoretical knowledge scores. Experimental: problem-based learning method. Control: traditional teaching method. CI = confidence interval, SD = standard deviation, SMD = standardized mean difference.

Nineteen studies reported on theoretical knowledge score results. There were 827 participants in PBL group and 843 participants in traditional teaching group. The influence diagnostics analysis showed WJ Hou, L Li, and Y Li are the 3 studies with most heterogeneity (Fig. 3A and see Supplementary Content). After excluding the study of WJ Hou, L Li, and Y Li, the meta-analysis results showed the PBL group significantly increased theoretical knowledge scores by a standardized mean of 3.17 compared with those of the traditional teaching model (95% CI: 2.28, 4.07, Fig. 4). However, the heterogeneity was still significant in the pooled effect (*I*^2^ = 98%, *P* < .01) and also subgroups.

### 3.5 Meta-analysis result for student satisfaction

**Figure 5:**
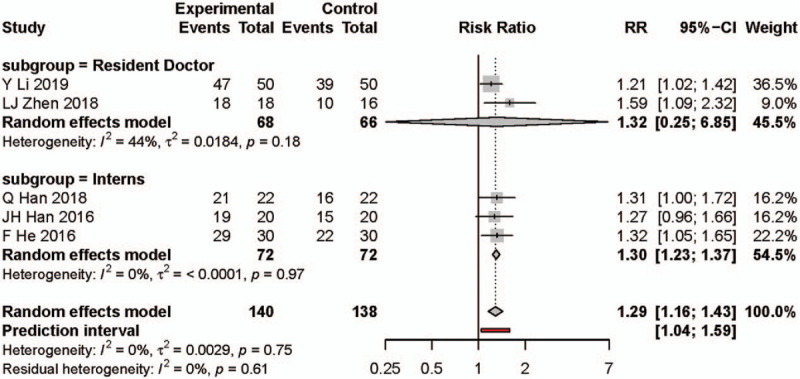
Forest plot and subgroups analysis results for student satisfaction. Experimental: problem-based learning method. Control: traditional teaching method. CI = confidence interval, RR = risk ratio.

A total of 8 studies reported on student satisfaction on a dichotomous scale. Two hundred thirty-nine participants were enrolled in the PBL group and 256 participants in the traditional teaching group. The studies conducted by XM Shen, YJ Zhang, and HJ Chen are the 3 studies with most heterogeneity (Fig. 3B and see Supplementary Content). After excluding these studies, the meta-analysis of the student satisfaction found that the PBL teaching model significantly increased student satisfaction compared with the traditional teaching model (risk ratio: 1.29, 95% CI: 1.16, 1.43, Fig. 5). There was insignificant heterogeneity in pooled effect (*I*^2^ = 0%, *P* = .75).

### 3.6 Meta-analysis result for clinical practice

**Figure 6:**
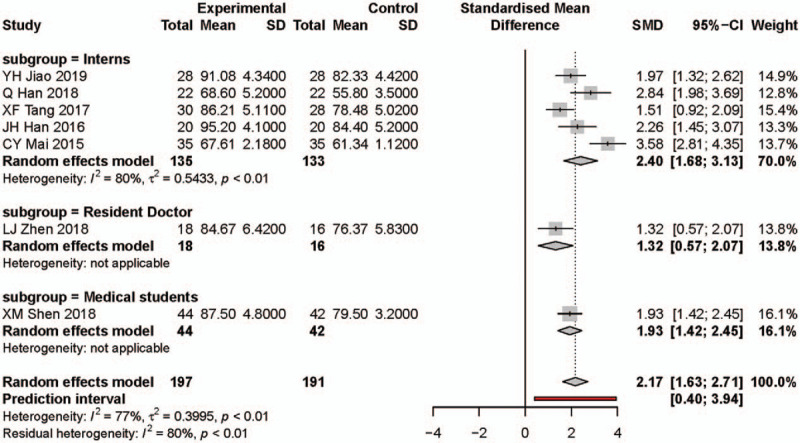
Forest plot and subgroups analysis results for clinical practice. Experimental: problem-based learning method. Control: traditional teaching method. CI = confidence interval, SD = standard deviation, SMD = standardized mean difference.

Ten studies reported their results regarding clinical practice. There were 318 participants in the PBL group and 312 participants in the traditional teaching group. The influence diagnostics analysis showed HJ Chen, YY Wang, and XL Wang are the 3 studies with most heterogeneity which we excluded before the meta-analysis (Fig. 3C and see Supplementary Content). Compared with the traditional teaching model, the PBL teaching model significantly increased the clinical practice score (SMD: 2.17, 95% CI: 1.63, 2.71, Fig. 6). The heterogeneity was significant across the pooled effect result (*I*^2^ = 77%, *P* < .01) and subgroup results.

### 3.7 Meta-analysis result for clinical operation

**Figure 7:**
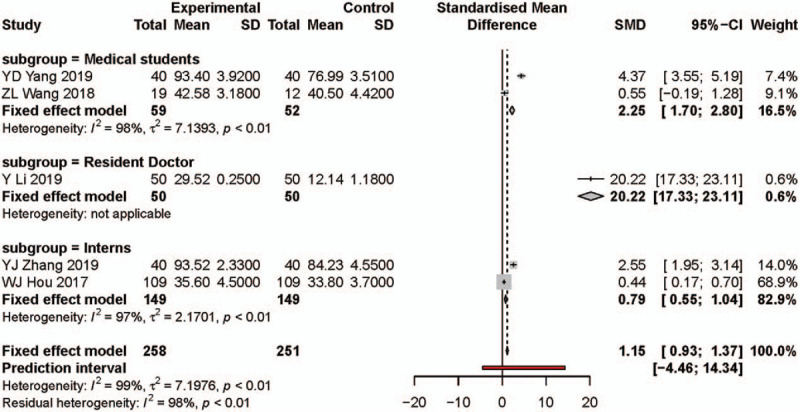
Forest plot and subgroups analysis results for clinical operation. Experimental: problem-based learning method. Control: traditional teaching method. CI = confidence interval, SD = standard deviation, SMD = standardized mean difference.

As for clinical practice scores, 5 studies reported on a continuous scale. Two hundred thirty-nine participants were enrolled in the PBL group and 256 the traditional teaching group, respectively. Since the rather small number of studies, we did not exclude any studies (Fig. 3D and see Supplementary Content). The meta-analysis results showed that the PBL teaching model increased clinical operation scores significantly compared with traditional teaching (SMD: 1.15, 95% CI: 0.93, 1.37, Fig. 7). Significant heterogeneity was found in the pooled effect (*I*^2^ = 98%, *P* < .01) and also subgroups.

## 4 Discussion

As compared with the traditional teaching model, 1 central idea of PBL is that the learning situation activates prior knowledge, facilitates learning new knowledge. It is resembling the ways in which knowledge will be demanded in real-world situations, through which students can increase the probability of recalling and applying what is stored in memory.^[59]^ This was supported by the results that the PBL group showed significant improvements in the theoretical knowledge exam compared with the traditional teaching group. However, when we interpret such a result, it should be noticed that there are many factors that affect the exam scores. Feeley et al^[60]^ asserted that important factors such as motivation, learning skills, and study methods should be taken into account which makes it difficult to make solid conclusions about the effect of PBL and traditional teaching method take in theoretical knowledge scores.

Moreover, the scores of clinical practices are based on items such as medical history collecting, physical examination, case presentation, and diagnosis. The assessment of clinical operation takes more emphasis on operation capability. For students in gynecology and obstetrics,^[61]^ it is important to not only be a theoretical knowledge learner, but also a clinical practitioner. Our study also showed better clinical practices and operation scores in the PBL group compared with traditional teaching group. These results were contrary to an earlier study conducted in England,^[62]^ which concluded that, no measurable difference observed in clinical evaluations comparing basic science education by traditional and PBL. Notably, a significant improvement of knowledge-related outcomes in PBL group were reported in several published articles globally.^[63–65]^ The discrepancy between the knowledge- and skill related outcomes in China and other countries could be explained by several factors. Firstly, given the fact that PBL model is such a novelty for most of Chinese students, it would stimulate their interest in learning^[66]^ greatly. Another point is that, in the PBL group, students^[67]^ usually would have more contacts with teachers. The habit of clinical thinking would probably be exercised more frequently.

Another crucial parameter of the effectiveness of one teaching method is student satisfaction. PBL has shown a consistent popularity among students in different courses,^[68]^ which is also the case in our study. In previous studies^[63,64]^ conducted specifically in obstetrics and gynecology courses in India and USA, researchers have demonstrated that, compared to the traditional teaching group, the PBL method resulted in better outcomes of critical thinking, problem solving skills, and greater learning satisfaction. Similarly, Sally et al^[66]^ showed that the PBL method was associated with improved student and faculty satisfaction. Several important points were reported to be the key players in achieving such popularity including small group size, realistic case scenarios.^[68]^ Organizers should focus on these factors when designing and constructing the courses. Other studies also showed PBL resulted in better outcomes such as communication skills, critical thinking, and passion for learning. The authors did not measure these outcomes since the number of studies is relatively small and a not reliable result could be obtained.

The heterogeneity of the current study is one non-negligible concern when interpreting our results. Interestingly, the heterogeneity did not significantly alter after influence diagnosis and subgroup analysis, except the teaching satisfaction which heterogeneity of pooled effect could be partially explained by the subgroup stratification. However, the comparatively small number of studies in most of the subgroups and large credential interval makes it hard to draw solid conclusion within subgroups. Many factors could contribute to heterogeneity. Firstly, the methodologies to implement the PBL were not unified in China, such as the time distribution of each procedure in PBL. Second, the organizers who are actually teaching students would be another potential contributor to the heterogeneity since the learning process could be seriously impacted by the teachers’ performances. The learning habit of students is also an important source that is hard to unify.

Other than the obvious heterogeneity, the current research has a few other limitations. For example, the current work is based on the RCTs which is restricted to China. Moreover, although, our study enrolled RCTs mostly, all these trials have an unclear bias in terms of blinding of participants and personnel and blinding of outcome assessment. This would inevitably undermine the methodological quality of our study.

To sum up, the current study focused on the effectiveness of PBL in obstetrics and gynecology education in China compared with the traditional teaching method. The results showed significant improvements in theoretical knowledge, student satisfaction, clinical practice, and clinical operation in the PBL group. Nonetheless, more delicate-designed studies on this topic are needed in the future to validate these results.

## Author contributions

**Conceptualization:** Jun Gu.

**Data curation:** Siwei Bi, Ruiqi Liu, Jingyi Li, Jun Gu.

**Formal analysis:** Siwei Bi, Ruiqi Liu, Jingyi Li, Jun Gu.

**Funding acquisition:** Siwei Bi, Ruiqi Liu, Jun Gu.

**Investigation:** Siwei Bi, Ruiqi Liu, Jingyi Li, Jun Gu.

**Methodology:** Siwei Bi, Ruiqi Liu, Jingyi Li, Jun Gu.

**Project administration:** Siwei Bi, Ruiqi Liu, Jun Gu.

**Resources:** Siwei Bi, Ruiqi Liu, Jun Gu.

**Software:** Siwei Bi, Ruiqi Liu, Jun Gu.

**Supervision:** Siwei Bi, Ruiqi Liu, Jun Gu.

**Validation:** Siwei Bi, Ruiqi Liu, Jingyi Li, Jun Gu.

**Visualization:** Siwei Bi, Ruiqi Liu, Jun Gu.

**Writing – original draft:** Siwei Bi, Ruiqi Liu.

**Writing – review & editing:** Siwei Bi, Ruiqi Liu, Jun Gu.

## Supplementary Material

Supplemental Digital Content
