# Supplementary material for: The effectiveness of problem-based learning in gynecology and obstetrics education in China: A meta-analysis of randomized controlled trials
Source: Medicine (Baltimore). 2021 Mar 5;100(9):e24660. doi: 10.1097/MD.0000000000024660 (PMC7939176; doi:10.1097/MD.0000000000024660)
Supplement: Supplemental Digital Content [file medi-100-e24660-s001.pdf]

The effectiveness of problem-based learning in gynecology and obstetrics education in China: a meta-analysis of randomized controlled trials

Siwei Bi[1#], Ruiqi Liu[1#], Jingyi Li[2], Jun Gu[3]*
1, Department of Burn and Plastic Surgery, West China Hospital, Sichuan University, Chengdu, Sichuan, 610041, People's Republic of China.
2 West China School of Medicine, Sichuan University, Chengdu, Sichuan, 610041, People's Republic of China.
3. Department of Cardiovascular Surgery, West China Hospital, Sichuan University, Chengdu, Sichuan, 610041, People's Republic of China.
# Authors contribute equally
* Corresponding to: Jun Gu, M.D.

Influence analysis for theoretical knowledge

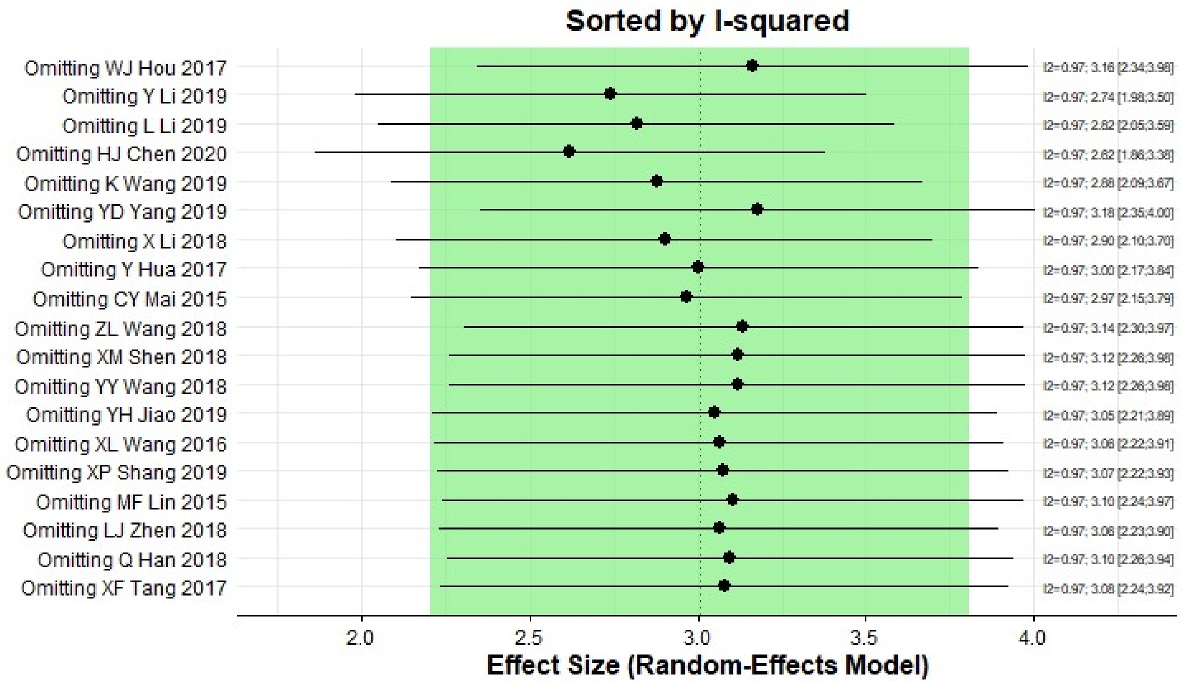

Figure S1. Influence analysis plots for theoretical knowledge score. WJ Hou, L Li and Y Li are the three studies with most heterogeneity

Influence for student satisfaction

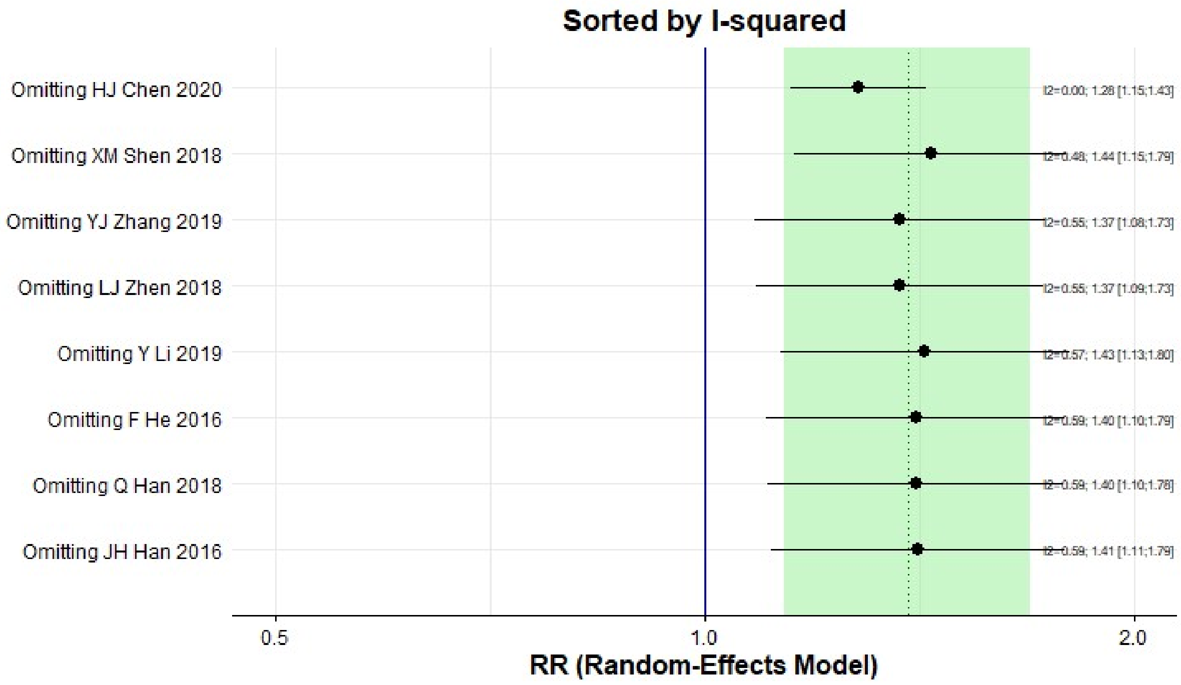

Figure S2. Influence analysis plots for student satisfaction. HJ Chen, XM Shen and YJ Zhang are the three studies with most heterogeneity. The order of forest plot were sorted by I^2 values.

Influence analysis for clinical practice

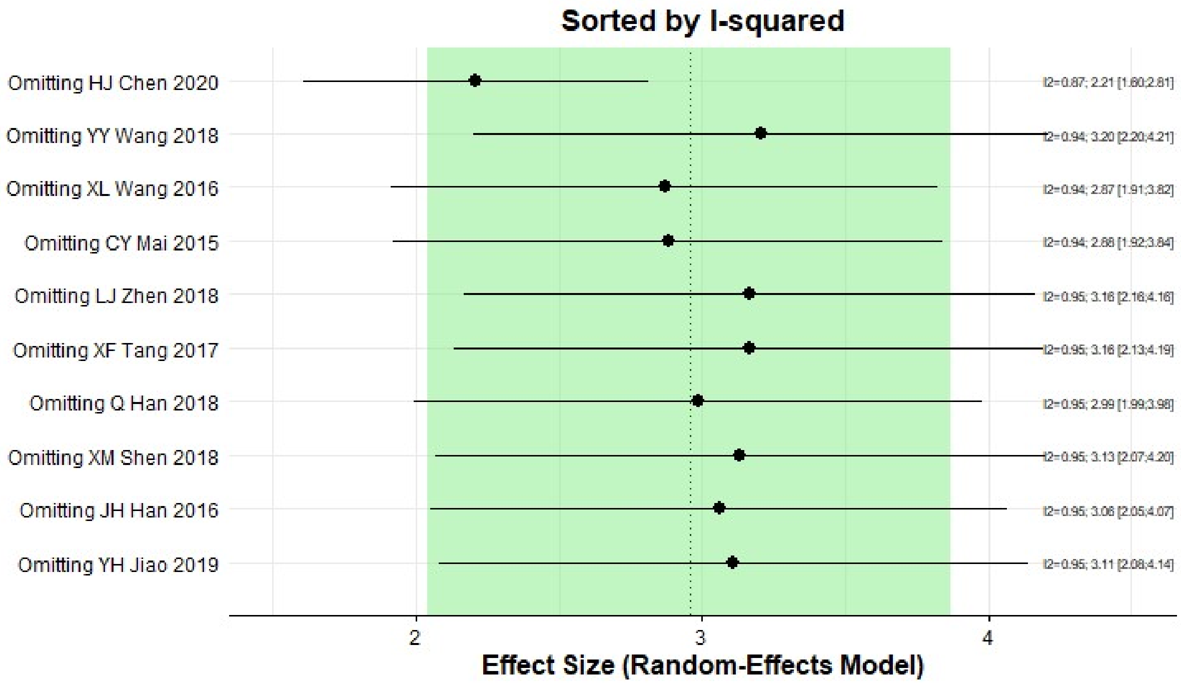

Figure S3. Influence analysis plots for clinical practice. HJ Chen, YY Wang and XL Wang are the three studies with most heterogeneity. The order of forest plot were sorted by I^2 values.

Influence analysis for clinical operation

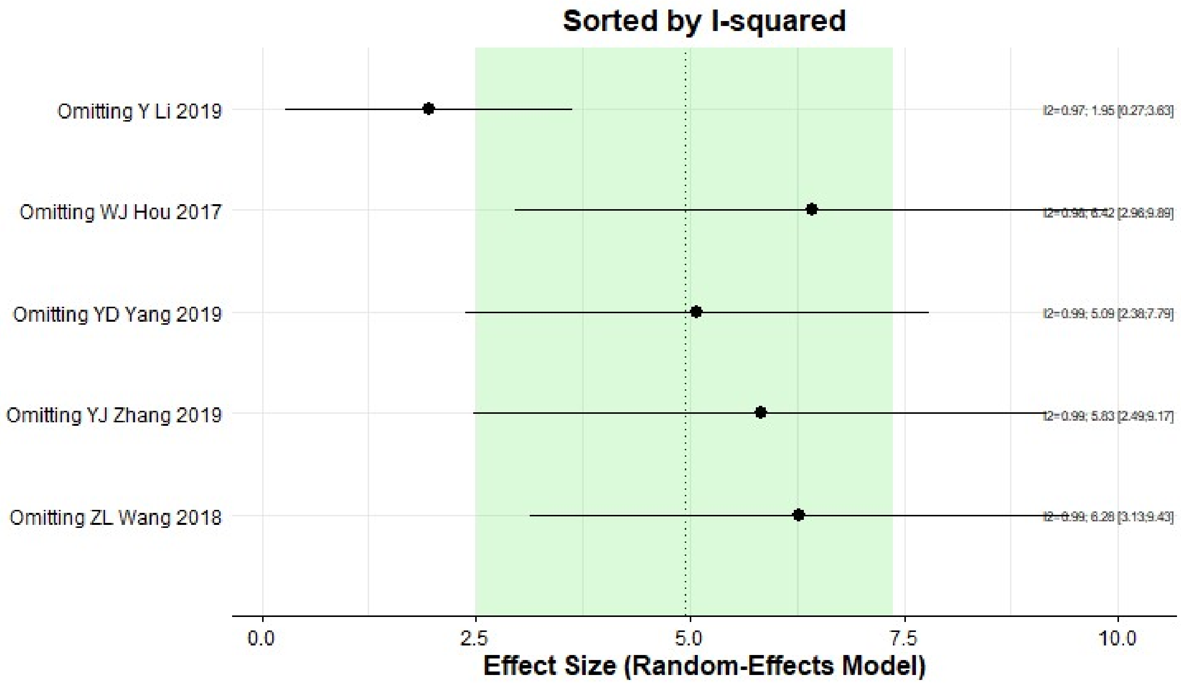

Figure S4. Influence analysis plots for clinical operation. The order of forest plot were sorted by I^2 values.
